# Systematic expression analysis of WEE family kinases reveals the importance of PKMYT1 in breast carcinogenesis

**DOI:** 10.1111/cpr.12741

**Published:** 2019-12-14

**Authors:** Yu Liu, Jian Qi, Zhen Dou, Jiliang Hu, Li Lu, Haiming Dai, Hongzhi Wang, Wulin Yang

**Affiliations:** ^1^ Anhui Province Key Laboratory of Medical Physics and Technology Center of Medical Physics and Technology Hefei Institutes of Physical Science Chinese Academy of Sciences Hefei China; ^2^ University of Science and Technology of China Hefei China; ^3^ Hefei Cancer Hospital Chinese Academy of Sciences Hefei China; ^4^ Hefei National Science Center for Physical Sciences at the Microscale University of Science and Technology of China Hefei China; ^5^ Department of Neurosurgery The Shenzhen People’s Hospital (The Second Clinical Medical Collage of Jinan University) Shenzhen China; ^6^ Department of Anatomy Shanxi Medical University Taiyuan China

**Keywords:** breast cancer, G2, M checkpoint, PKMYT1, PLK1, Prognostic biomarker, WEE family kinases

## Abstract

**Objectives:**

Many cancer cells depend on G2 checkpoint mechanism regulated by WEE family kinases to maintain genomic integrity. The PKMYT1 gene, as a member of WEE family kinases, participates in G2 checkpoint surveillance and probably links with tumorigenesis, but its role in breast cancer remains largely unclear.

**Materials and Methods:**

In this study, we used a set of bioinformatic tools to jointly analyse the expression of WEE family kinases and investigate the prognostic value of PKMYT1 in breast cancer.

**Results:**

The results indicated that PKMYT1 is the only frequently overexpressed member of WEE family kinases in breast cancer. KM plotter data suggests that abnormally high expression of PKMYT1 predicts poor prognosis, especially for some subtypes, such as luminal A/B and triple‐negative (TNBC) types. Moreover, the up‐regulation of PKMYT1 was associated with HER2‐positive (HER2+), basal‐like (Basal‐like), TNBC statuses and increased classifications of Scarff, Bloom and Richardson (SBR). Co‐expression analysis showed PKMYT1 has a strong positive correlation with Polo‐like kinase 1 (PLK1), implying they may cooperate in regulating cancer cell proliferation by synchronizing rapid cell cycle with high quality of genome maintenance.

**Conclusions:**

Collectively, this study demonstrates that overexpression of PKMYT1 is always found in breast cancer and predicts unfavourable prognosis, implicating it as an appealing therapeutic target for breast carcinoma.

## 1 INTRODUCTION

Malignant tumours are the most threatening human diseases around the world. In 2018, there were about 18.1 million new cancer cases and 9.6 million cancer‐related deaths.^1^ Among them, breast cancer is the most commonly diagnosed cancer and the leading cause of cancer‐related death among women. The incidence of this aggressive disease remains alarmingly high with more than one million newly diagnosed cases each year.^1^, ^2^, ^3^ Understanding the molecular mechanisms of breast carcinogenesis is an important task for researchers to develop new methods for diagnosis and treatment of this malignancy. Despite years of research, the overall 5‐year survival rate for patients with breast cancer remains low.^4^, ^5^ Therefore, there is still an urgent need for finding reliable biomarkers for early diagnosis, accurate prognosis and targeted therapy.^6^

During cell cycle, normal cells maintain the stability of the genome primarily through the DNA damage checkpoints, a surveillance mechanism that is frequently deregulated in cancers. Because of the loss‐of‐function of tumour suppressor genes, such as mutations in p53 that leads to the inactivation of the G1 checkpoint, many cancerous cells heavily rely on G2/M checkpoint to ensure its genomic stability and survival advantage. The WEE kinase, consisting of three family members in human, including PKMYT1 (membrane‐associated tyrosine‐ and threonine‐specific cdc2‐inhibitory kinase) and two WEE1 kinases (WEE1 and WEE1B), is protein kinase that activate the G2/M checkpoint of the cell cycle in response to double‐stranded DNA breaks.^7^, ^8^ Early study has shown that WEE1 inhibitors are effective against TP53‐mutant cancer cells, which account for over 80% of triple‐negative breast cancer (TNBC) cases.^9^

PKMYT1 is essential for Golgi and endoplasmic reticulum assembly in mammalian cells. It has been shown to be involved in G2 arrest in oocytes and its activity is regulated by AKT phosphorylation.^10^ PKMYT1 localizes to the cytoplasm by binding to the cell division cycle 2 (CDC2)/cyclin B complex.^11^ Its proposed function is to phosphorylate the Thr14/Thr15 residue on CDC2, thus inhibiting CDC2 activity and preventing cell cycle from entering mitosis.^12^, ^13^ Since PKMYT1 and WEE1 safeguard the G2/M phase transition, inhibitors against PKMYT1 and WEE1 may effectively lower the survival ability of tumour cells and thus hold therapeutic potential for clinical use. Previous studies have found that WEE1 inhibitor renders apoptosis in TNBC cells, but its clinical application remains limited.^9^, ^14^, ^15^ In other aspect, the role of PKMYT1 in breast cancer development remains unknown and awaits further investigations. In this work, we applied a wide range of integrated bioinformatics approach to assess the importance of PKMYT1 by analysing the expression, potential function and prognostic impact of PKMYT1 in human breast cancer.

## 2 MATERIALS AND METHODS

### 2.1 Data mining in Oncomine database

The Oncomine database (https://www.oncomine.org/resource/login.html) is a publicly accessible, online cancer microarray database that helps facilitate research from genome‐wide expression analysis. We used the Oncomine database to determine the transcription level of the PKMYT1 gene in breast cancer^16^, ^17^ by retrieving expression levels of PKMYT1 mRNA (log2‐transformed) in breast cancer vs normal tissues for statistical comparison. To obtain the most important PKMYT1 probe, the thresholds were set as follows: *P*‐value < 1E‐4, fold change >2 and the gene ranks in the top 10%.

### 2.2 University of California Santa Cruz (UCSC) cancer genomics browser analysis

The UCSC Cancer Genomics Browser (http://xena.ucsc.edu/) 18, 19 was used to verify the heat map of PKMYT1 expression, and the correlation between PKMYT1 and hub genes expression were analysed.

### 2.3 Catalogue of somatic mutations in cancer (COSMIC) analysis for PKMYT1 mutations

The COSMIC database (http://cancer.sanger.ac.uk) is a high‐resolution resource for studying the effects of somatic mutations in all forms of human tumours. We used this database to analyse mutations in PKMYT1 in breast cancer.^20^, ^21^ An overview of the distribution and substitutions on the coding strand in breast cancer was depicted in a pie chart.

### 2.4 Breast Cancer Gene‐Expression Miner v4.0 (bc‐GenExMiner v4.0)

The expression of PKMYT1 and its prognostic value in breast cancer were evaluated using Breast Cancer Gene‐Expression Miner v4.0 online dataset (http://bcgenex.centregauducheau.fr), which is a statistical mining tool that contains published annotated genomic data, including 36 annotated genomic datasets and 5861 patients with breast cancer.^22^, ^23^ Correlation between PKMYT1 and PLK1 genes was estimated by Pearson's correlation module of bc‐GenExMiner v4.0.

### 2.5 cBioPortal database analysis

Cancer genomics analysis was performed by querying the online cBioPortal for Cancer Genomics (bib24%7Cbib25://www.cbioportal.org/).^24^, ^25^ The cBioPortal for Cancer Genomics is attached to the Memorial Sloan Kettering Cancer Center and provides comprehensive analyses of complex tumour genomics and clinical profiles from research into 105 cancer types in The Cancer Genome Atlas (TCGA) (study ID, brca_tcga_pub2015). Using cBioPortal, we investigated the genes that are positively associated with PKMYT1 expression in breast cancer and the RNA sequencing data with the default setting by The Cancer Genome Analysis group (https://cancergenome.nih.gov/).

### 2.6 Gene correlation analysis in GEPIA

The online database Gene Expression Profiling Interactive Analysis (GEPIA) (http://gepia.cancer-pku.cn/index.html) 26 is an interactive web that includes 9736 tumours and 8587 normal samples from TCGA and the GTEx projects, which analyse the RNA sequencing expression. GEPIA based on gene expression with the log‐rank test and the Mantel‐Cox test in 33 different types of cancer. Gene expression correlation analysis was performed for given sets of TCGA expression data. The Spearman method was used to determine the correlation coefficient. PKMYT1 was presented on the x‐axis, and other genes of interest were represented on the y‐axis for tumour vs normal tissue analysis.

### 2.7 Search Tool for Retrieving Interacting Genes by STRING server

In this study, the STRING database (http://string-db.org) 27 was employed to construct a PPI network of co‐expressed genes with an interaction score of >0.4. Cytoscape (version 3.4.0) is an open source bioinformatics software platform for visualizing molecular interaction networks.^28^ Cytoscape's plug‐in Molecular Complex Detection (MCODE) (version 1.4.2) is an APP for clustering a given network based on topology to find tightly connected regions. The PPI network was drawn using Cytoscape, and the most important module in the PPI network was identified by MCODE. The selection criteria were as follows: MCODE score > 5 points, degree cut‐off = 2, node score cut‐off = 0.2, Max depth = 100, and k‐Score = 2.

### 2.8 Functional and KEGG Pathway Enrichment Analysis

DAVID (http://david.abcc.ncifcrf.gov/) is a functional annotation tool that reveals the biological significance behind by entering a list of genes.^29^, ^30^ Based on the extracted co‐expressed genes, GO analysis can be divided into three categories: biological processes (BP), cellular components (CC) and molecular functions (MF).^31^ The KEGG pathway database is used to identify biological pathways for co‐expressed gene enrichment.^32^ Statistical significance was assessed using Fisher's exact test, and *P*‐value < .05 was considered significant.

### 2.9 Statistical analysis

All statistical analyses were performed by default as described by web resources. Briefly, Students’ *t* test was conducted to compare mRNA expression in Oncomine database. Log‐rank test was used for computing *P*‐value in Kaplan‐Meier (KM) plotter. GEPIA differential analysis was tested using one‐way ANOVA by defining the disease state (Tumour or Normal) as variable. In DAVID annotation system, Fisher's exact test was adopted to measure the gene enrichment in annotation terms. In Breast Cancer Gene‐Expression Miner v4.0, the linear dependence (correlation) between two variables was measured using Pearson's correlation coefficient. The correlation of gene expression in cBioPortal and UCSC databases was evaluated by Spearman's correlation. *P* < .05 was considered to be statistically significant (*, *P* < .05; **, *P* < .01; ***, *P* < .001).

## 3 RESULTS

### 3.1 Up‐regulation of PKMYT1 mRNA expression in human breast cancer

**Figure 1:**
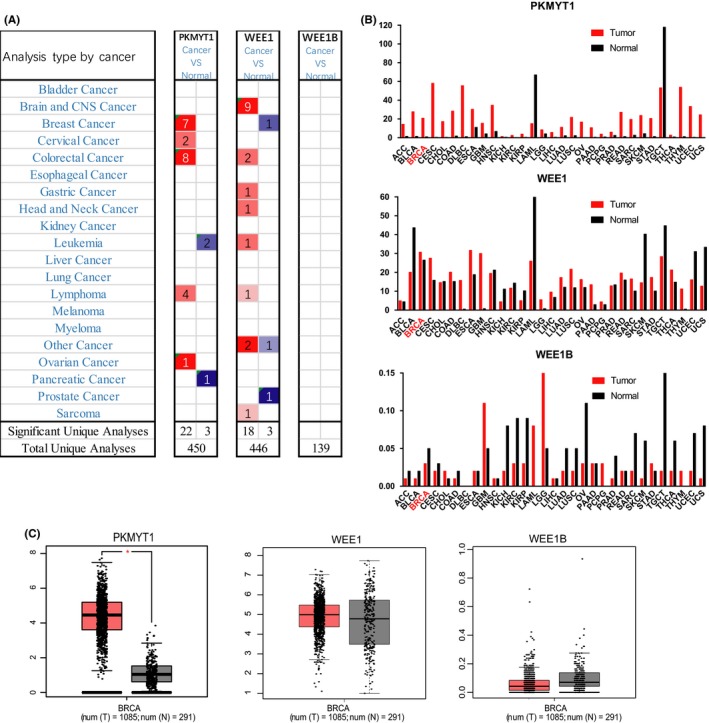
PKMYT1 mRNA expression was elevated in human breast cancer. A, This graph generated by Oncomine indicates the numbers of datasets with statistically significant mRNA overexpression (red) or downexpression (blue) of PKMYT1, WEE1 and WEE1B (cancer tissues vs corresponding normal tissues). The threshold was defined with the following parameters: *P*‐value of 1E‐4, fold change of 2 and gene ranking of 10%. B, C, The GEPIA database verified that PKMYT1 gene expression was significantly upregulated in breast cancer tissues (BRCA) (n = 1085) compared with normal breast tissues (n = 291), *P < .05

**Figure 2:**
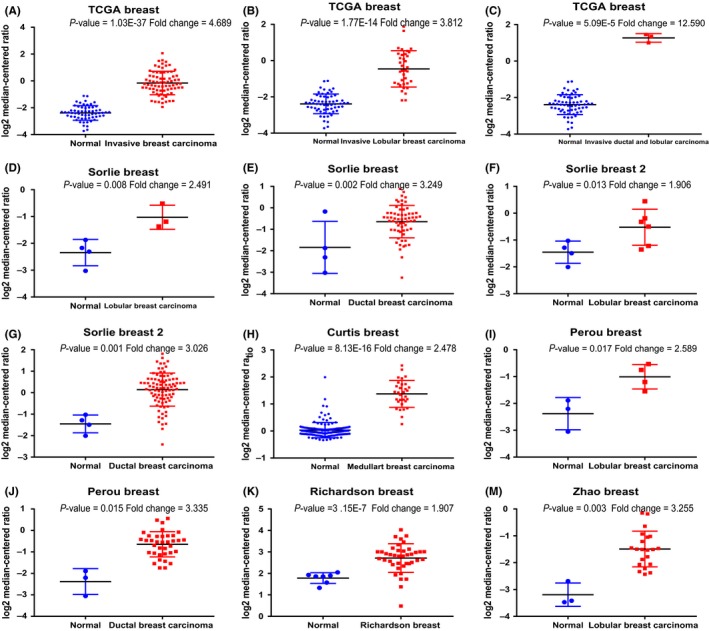
Analysis of PKMYT1 gene expressions in different subtypes of breast cancer using the Oncomine database. Box plot derived from gene expression data in the Oncomine database comparing the expressions of PKMYT1 between normal tissues and cancer tissues in different subtypes of breast cancer, including invasive breast carcinoma, invasive ductal breast carcinoma, mixed lobular and ductal breast carcinoma, invasive lobular breast carcinoma, intraductal cribriform breast adenocarcinoma, and invasive ductal and lobular carcinoma

We analysed the expression profile of WEE family kinases using Oncomine database. The expression of PKMYT1, but not of WEE1 and WEE1B, was significantly elevated in several solid tumours, especially in breast cancer and colorectal cancer (Figure 1A). The mining of GEPIA database further confirmed that PKMYT1 was the only member of WEE family kinases unregulated in breast cancer (BRCA) tissues in relative to normal tissues (Figure 1B,C). Furthermore, Oncomine analysis of cancer vs normal samples in different patient datasets revealed that PKMYT1 expression was significantly higher in invasive breast carcinoma, invasive lobular breast carcinoma, invasive ductal breast carcinoma, male breast carcinoma, medullary breast carcinoma, mucinous breast carcinoma, ductal breast carcinoma in situ and tubular breast carcinoma (Figure 2) (Table 1).

**Table 1:** PKMYT1 expressions are upregulated in different subtypes of breast carcinoma

Subtype of breast cancer	*P*‐value	FC	Rank (%)	Sample	Reference
Invasive Breast Carcinoma	1.03E‐37	4.689	1	137	TCGA
Invasive Lobular Breast Carcinoma	1.77E‐14	3.812	2	97	TCGA
Invasive Ductal Breast Carcinoma	3.46E‐53	4.827	1	450	TCGA
Invasive Ductal and Lobular Carcinoma	5.09E‐05	12.59	4	64	TCGA
Invasive Lobular Breast Carcinoma	1.77E‐14	3.812	2	97	TCGA
Medullary Breast Carcinoma	8.13E‐16	2.478	1	176	TCGA
Ductal Breast Carcinoma in Situ	.015	3.335	10	39	TCGA
Intraductal Cribriform Breast Adenocarcinoma	3.03E‐07	4.347	2	64	TCGA
Mixed Lobular and Ductal Breast Carcinoma	1.23E‐05	3.076	2	68	TCGA
Lobular Breast Carcinoma	.017	2.589	3	7	TCGA
FC, Fold Change					

### 3.2 PKMYT1 mutations are rare and high PKMYT1 expression predicts poor prognosis in breast cancer

**Figure 3:**
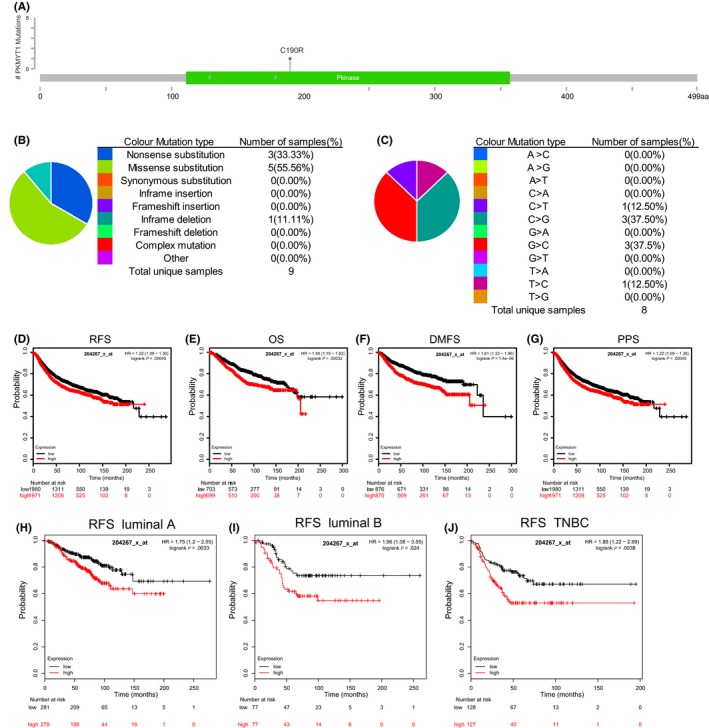
PKMYT1 mutations and prognostic significance in human breast cancer. A, Schematic representation of PKMYT1 mutations (TCGA) using the cBioportal. B, C, The percentages of mutation types of PKMYT1 in breast cancer were indicated in a pie chart generated from Catalogue of Somatic Mutations in Cancer database. D‐J, Prognostic significances of PKMYT1 gene expression in patients with breast cancer were shown based on the KM plotter database. RFS, relapse‐free survival; OS, overall survival; DMFS, distance metastasis‐free survival; PPS, post‐progression survival; and HR, hazard ratio

We employed cBioPortal to evaluate the frequency of changes in PKMYT1 mutations in breast cancer. The frequency of mutation is very low, only 0.1% (Figure 3A). The mutations of PKMYT1 in breast cancer were analysed using the COSMIC database. The pie chart describes the types of mutations, including nonsense mutations, missense mutations, and in‐frame deletions, the largest proportion of which are missense mutations, up to 55.56% (Figure 3B). Nucleotide changes included C > T, C > G, G > C and T > C mutations, with the largest proportion being C > G and G > C (Figure 3C). Using the Kaplan‐Meier (KM) plotter as an indicator of prognostic value of PKMYT1 expression, we found that increased expression of PKMYT1 mRNA was significantly associated with overall survival (OS), post‐progression survival (PPS), relapse‐free survival (RFS) and distant metastatic‐free survival (DMFS) (Figure 3D‐G). Depending on the molecular characteristic, breast cancers can be further divided into several subtypes, including luminal epithelial type (luminal type), HER2 overexpression type and basal type (three negative type, normal breast type cell type),^33^ which could vary for the prognosis and adjuvant treatments. Looking into the relationship between PKMYT1 and breast cancer subtypes, we found that RFS was highly affected by the expression levels of PKMYT1 as shown by KM plotter analysis. It appears that the higher the expression of PKMYT1, the shorter the survival period in luminal A, B and TNBC subtypes (Figure 3H‐J), suggesting that PKMYT1 may be a reliable biomarker for breast cancer prognosis.

### 3.3 The associations of PKMYT1 expression profiles and clinical parameters in breast cancer patients

**Figure 4:**
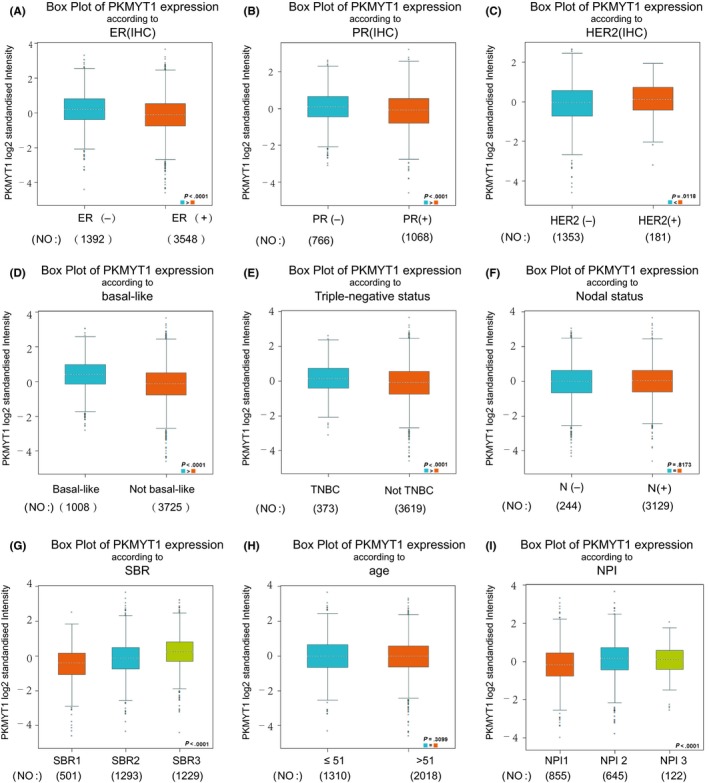
Associations between PKMYT1 gene expressions and clinical‐pathological parameters in breast cancer. Notable global differences between the groups were evaluated by Welch's *t* test. A, ER status, oestrogen receptor; (B) PR status, progesterone receptor; (C) HER2 status, human epidermal growth factor receptor 2; (D) Basal‐like status; (E) triple‐negative status; (F) nodal status; (G) SBR status; (H) age status; (I) NPI status

The expression profiles of PKMYT1 were examined across PAM50 breast cancer subtypes using 5861 patients with breast cancer cohorts in bc‐GenExMiner 4.0, based on different clinical‐pathological indicators; estrogen receptors group and progesterone receptors groups were compared with the corresponding positive groups. PKMYT1 mRNA expression was significantly increased in the body of ER‐ and PR‐groups, (*P* < .0001), (Table 2 and Figure 4A,B). However, compared with HER2+, HER2‐ patients had somewhat decrease in PKMYT1 mRNA levels with *P*‐value of 0.0118 (Figure 4C). In addition, patients with Basal‐like status showed significantly increased PKMYT1 expression (*P* < .0001) compared with patients with negative Basal‐like status (Table 2 and Figure 4D). Compared with non‐TNBC group, PKMYT1 mRNA expression was significantly higher in TNBC patients (*P* < .0001) (Table 2 and Figure 4E), but not in the case with Nodal Status (*P* = .8173) (Table 2 and Figure 4F). In the Scarff, Bloom and Richardson (SBR) grade^34^ status criteria, increased SBR levels were significantly associated with increased PKMYT1 transcript levels in relative to the SBR1 group (*P* < .0001) (Figure 4G). There was no significant relationship between ages (*P* = .3099) (Figure 4H). With higher rate of Nottingham Prognostic Index (NPI) classification, the lower of the survival rate was associated (Figure 4I).

**Table 2:** The associations of PKMYT1 expressions with clinical manifestations in breast carcinoma

**Variables**	No*	PKMYT1	*P*‐value
Age			
≤51	1310	‐	*P* = .3099
>51	2018	‐	
Nodal status			
−	2351	‐	*P* = .8173
+	1440	‐	
ER			
−	1392	↑	<.0001
+	3548	‐	
PR			
−	766	↑	<.0001
+	1068	‐	
HER2			
−	1353	‐	*P* = .0118
+	181	↑	
Basal‐like Status			
Not	3725	‐	<.0001
Basal‐like	1008	↑	
Triple‐negative Status			
NOT	3619	‐	<.0001
TNBC	373	↑	

Abbreviations: ↑, upregulated; ER, oestrogen receptor; HER2, human epidermal growth factor receptor 2; PR, progesterone receptor; TNBC, triple‐negative breast cancer.

### 3.4 KEGG and GO enrichment analysis revealing functional association of PKMYT1 with cell proliferation

**Figure 5:**
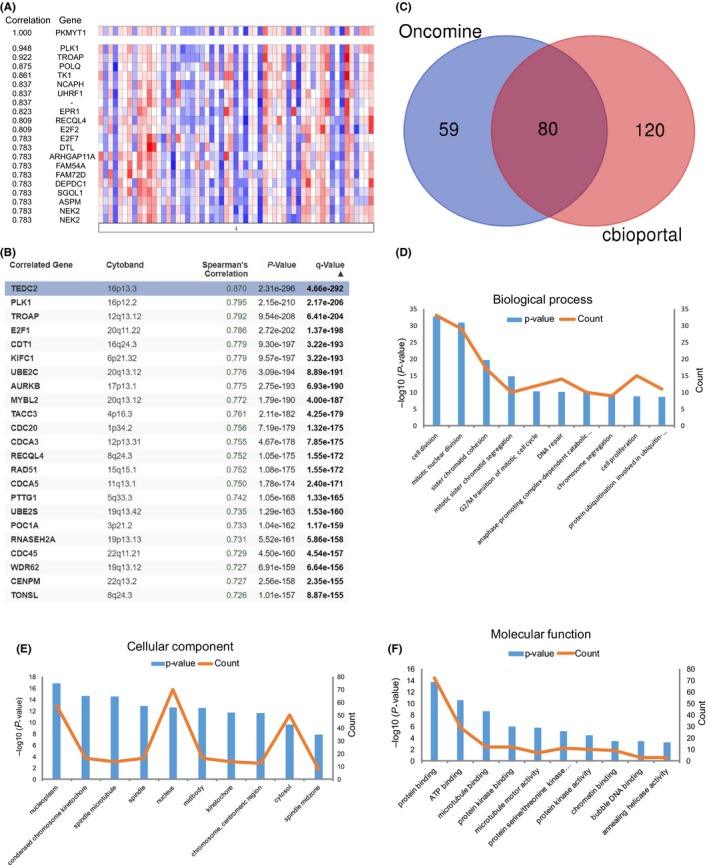
KEGG and GO enrichment analyses of co‐expressed genes indicating an association of PKMYT1 with cell proliferation. A, The top 150 genes in breast cancer positively associate with PKMYT1 transcript level based on the Oncomine database (Stickeler Breast dataset) [correlation ≥0.638 (log2 median‐centred ratio)]. By removing 11 gene duplications, 139 genes were finally used. B, The top 200 genes positively associate with PKMYT1 transcript level based on the GEPIA database with breast cancer (TCGA provisional, 1105 samples) (Spearman's correlation ≥ 0.561, *P*‐value ≤ 1.54e‐80). C, Venn diagram represents the intersection of top positively corrected genes between the Oncomine database and the GEPIA database. D, GO enrichment of co‐expressed genes in biological process, (E) cellular component and (F) molecular function

The Oncomine database (Stickeler Breast dataset) (Figure 5A) was used to select the top 150 co‐expressed genes of PKMYT1 [Correlation ≥ 0.638 (log2 median‐centred ratio)]. Meanwhile the cBioPortal dataset (Figure 5B) was applied to obtain top 200 co‐expressed genes (Spearman's correlation ≥ 0.561, *P*‐value ≤ 1.54e‐80) for Breast invasive carcinoma (TCGA, provisional, 1105 samples). The co‐expressed genes obtained from the two databases were cross‐referenced to obtain a cohort of 80 common co‐expressed genes (Figure 5C). To analyse the biological classification of co‐expressed genes, we used DAVID tool for functional and pathway enrichment analysis. GO analysis indicated that the biological processes including cell division, mitotic nuclear division, sister chromatid cohesion, mitotic sister chromatid segregation and G2/M transition of mitotic cell cycle were significantly affected (Figure 5D), consistent with enrichment in respective cellular locations and proposed molecular functions (Figure 5E,F) (Table 3). Collectively, these data suggest an essential role of PKMYT1 in regulating cell proliferation in breast cancer.

**Table 3:** KEGG enrichment analysis of co‐expressed genes with PKMYT1

Term	Description	Count in gene set	*P*‐value
hsa04110	Cell cycle	18	1.29334E‐21
hsa04114	Oocyte meiosis	11	7.53192E‐11
hsa04914	Progesterone‐mediated oocyte maturation	8	1.65385E‐07
hsa04115	p53 signalling pathway	6	1.68503E‐05
hsa03460	Fanconi anaemia pathway	5	.00012275
hsa05166	HTLV‐I infection	7	.001344751
hsa03440	Homologous recombination	3	.008855926
hsa05161	Hepatitis B	4	.03408854
hsa05212	Pancreatic cancer	3	.040627727
hsa05206	MicroRNAs in cancer	5	.050949963
hsa05222	Small cell lung cancer	3	.06561741
hsa05203	Viral carcinogenesis	4	.079470245

### 3.5 PKMYT1 PPI network construction and analysis of 10 hub genes

**Figure 6:**
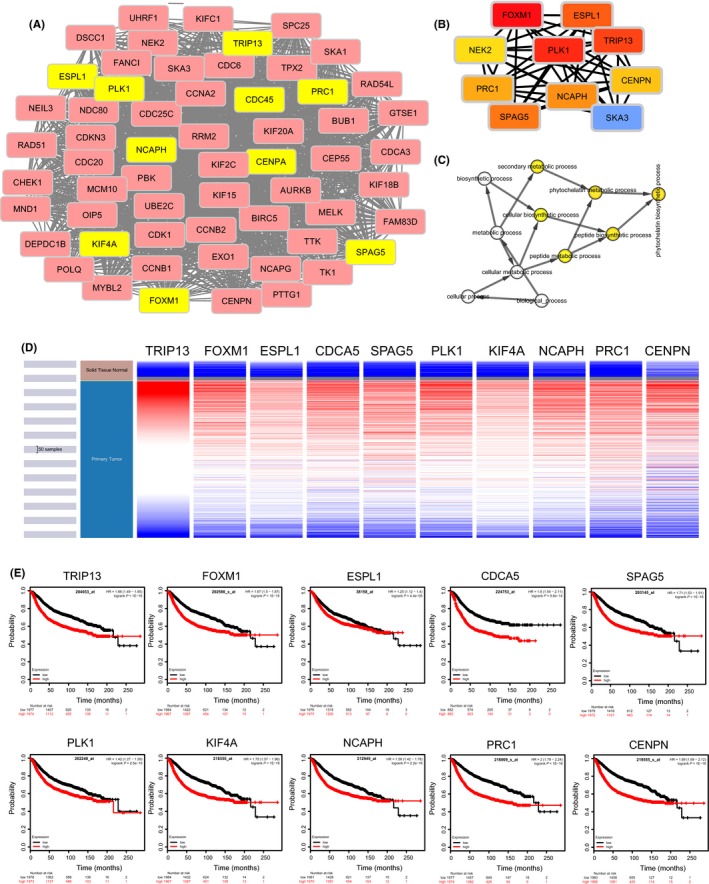
Construction of PPI network of PKMYT1 positive‐correlation genes and analysis of hub genes. The most significant modules and hub genes of the PPI network were analysed by Cytoscape software. A, Clustering analysis of PKMYT1 co‐expressed genes by STRING tools. B, The hub genes were identified using cytoHubba tool kits in Cytoscape. C, The biological process analysis of hub genes was performed using the BiNGO plug‐in. *P* < .05 was considered to be a statistically significant difference. D, The hierarchical clustering of hub genes was constructed using UCSC online database. E, Over survival analyses of hub genes in breast cancer. The results based on the KM plotter database indicate all hub genes are associated with poor prognosis in breast cancer

Using the STRING database, the co‐expressed 80 genes were constructed into a protein‐protein network, and the most important module was obtained using Cytoscape (MCODE plug‐in) (Figure 6A). The top ten genes, including PLK1, NCAPH, TRIP13, KIF4A, SPAG5, CDCA5, FOXM1, ESPL1, PRC1 and CENPN, were identified as potential hub genes according to the degree score generated by CytoHubba plug‐in (the cytoHubba plug‐in, top 10 nodes ranked by DMNC) (Figure 6B), consistent with their enrichment in the top module analysed by MCODE (highlighted in yellow) (Figure 6A). The biological process analysis of hub genes was further performed using BINGO plug‐in. Particularly, peptide biosynthetic process, phytochelatin biosynthetic process, cellular biosynthetic process, peptide metabolic process, secondary metabolic process and phytochelatin metabolic process were largely altered, suggesting that they may participate in the protein anabolism required for cell division (Figure 6C). Hierarchical clustering of the hub genes was performed using UCSC Cancer Genomics Browser (Figure 6D), indicating the concordant expression pattern across 10 genes. Furthermore, the overall survival of hub genes was analysed using Kaplan‐Meier curve. All these 10 hub genes exhibited poorer overall survival rate in higher expression groups (Figure 6E). Amongst these hub genes, PLK1 may be the most attractive target in cell proliferation. A large number of studies have shown that PLK1 is one of the serine‐threonine kinase families highly expressed in prostate cancer,^35^ neuroblastoma cells,^36^ acute myeloid leukaemia,^37^ cervical cancer^38^ and other malignant tumours, which plays an important role in the initiation, maintenance and completion of mitosis. Interestingly, PLK1 has been proposed to be the functional partner of PKMYT1 in regulating cell cycle,^7^, ^39^, ^40^ and PLK1 is also closely related to breast cancer,^41^ implying that PLK1 and PKMYT1 may play an cooperative role in the development of breast cancer.

### 3.6 Co‐expression of PKMYT1 and PLK1

**Figure 7:**
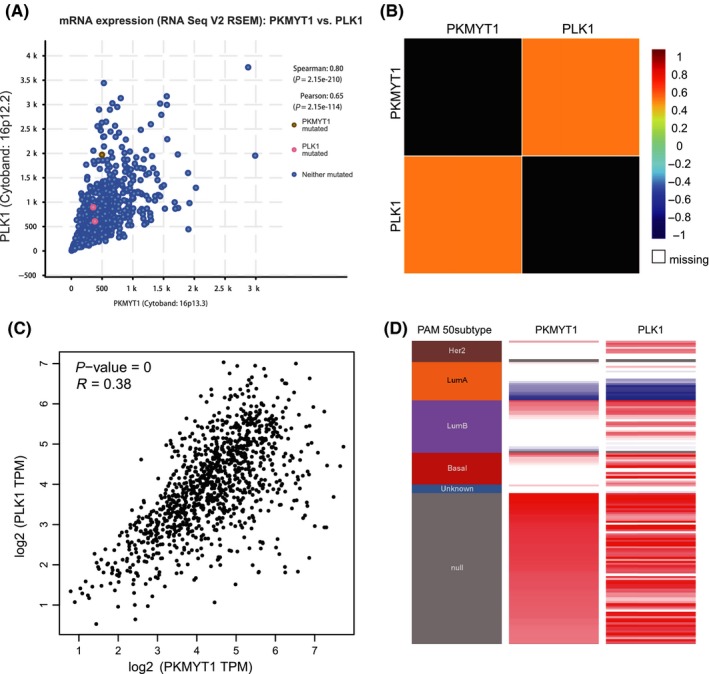
Expressions of PKMYT1 and PLK1 genes are highly correlated. A, The correlation between PKMYT1 and PLK1 co‐expression analysed using cBioportal. B, The relationship between PKMYT1 and PLK1 in breast cancer analysed using bc‐GenExMiner v4.0. C, Correlation between PKMYT1 and PLK1 mRNA expression determined using GEPIA. D, Heat map of PKMYT1 expression and PLK1 mRNA expression across PAM50 breast cancer subtypes in the TCGA database determined using UCSC Xena

cBioportal regression analysis showed that PKMYT1 and PLK1 had high correlation coefficients (Spearman's correlation = 0.79; Pearson's correlation = 0.60) (Figure 7A). This positive correlation between PKMYT1 and PLK1 transcript was substantiated by the analysis via both the bc‐GenExMiner 4.0 database (Figure 7B) and GEPIA (Figure 7C). This was further confirmed using UCSC Xena with consistent correlative patterns in different subtypes (Figure 7D). These data demonstrate that PKMYT1 has a strong association with PLK1, suggesting that they may be functional partners in breast carcinoma.

### 3.7 High PLK1 expression predicts unfavourable prognosis in patients with breast cancer

**Figure 8:**
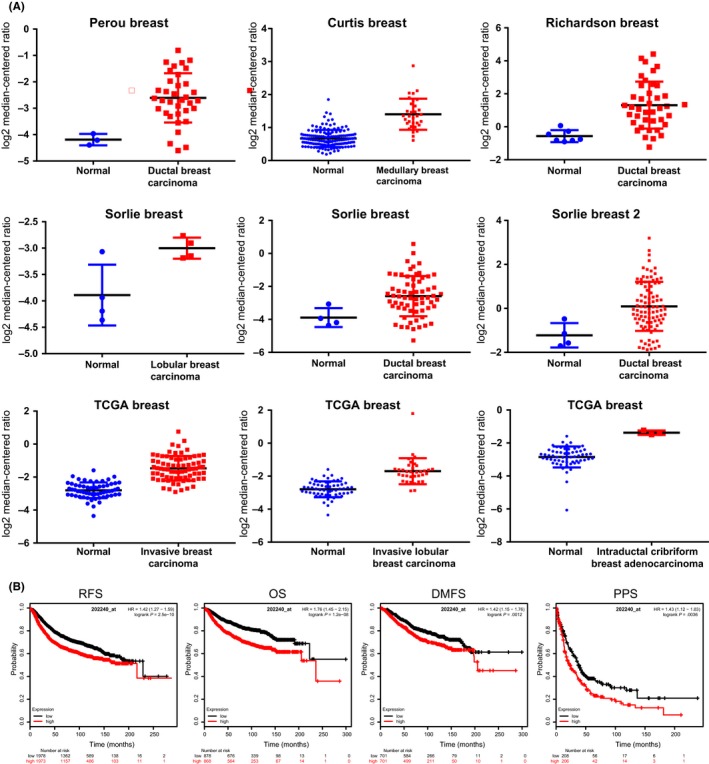
The expression of PLK1 is upregulated in breast cancer and associated with poor prognosis. A, Invasive breast carcinoma, invasive ductal breast carcinoma, mixed lobular and ductal breast carcinoma, invasive lobular breast carcinoma, intraductal cribriform breast adenocarcinoma, and invasive ductal and lobular carcinoma were included in the box plots derived from the Oncomine database. B, Survival analyses of PLK1 in breast cancer using KM plotter. OS, overall survival; RFS, relapse‐free survival; DMFS, distant metastasis‐free survival. PPS, post‐progression survival

To determine the genetic alteration of PLK1 in breast cancer, the expression profile of PLK1 was investigated using the Oncomine database. PLK1 expression was found to be upregulated in almost all different subtypes of breast cancer by analy'sing a wide range of dataset, including invasive ductal and invasive lobular breast cancer, tubular breast cancer, invasive lobular breast cancer, mucinous breast cancer, invasive ductal breast cancer and mixed lobular and mammary glands (Figure 8A). Subsequently, the prognostic value of PLK1 in breast cancer was studied by Kaplan‐Meier plotter database, and it was confirmed that high expression of PLK1 mRNA was significantly associated with the decrease of RFS, OS, DMFS and PPS in breast cancer (Figure 8B).

## 4 DISCUSSION

Breast cancer is one of the most common malignancies in the middle‐aged and elderly women worldwide, with over one million breast cancers occurring every year worldwide.^1^, ^42^ Despite significant progress in breast cancer treatment in recent years, the challenges in curing this disease have not been fully addressed. Research on the pathogenesis, diagnosis and treatment of breast cancer remains an area of active investigation.^43^

This study was the first to investigate the mRNA expression and prognosis of PKMYT1 in breast cancer, although other studies have reported PKMYT1 alternations in the occurrence and development of several cancers, including liver^44^ and colorectal carcinomas.^45^ As the key regulators of G2/M transition, WEE family kinases play essential role in maintaining cell genomic stability under rapid cell proliferation. Our study has revealed that PKMYT1 is the only overexpressed member of WEE family kinases in breast cancer tissues, suggesting its predominant role in monitoring G2/M transition in breast cancer cell division. Through our analysis, PKMYT1 expression levels were significantly correlated with ER‐, PR‐, HER2+, Basal‐like status and TNBC subtypes, consistent with the indication of poor prognosis in patients with breast cancer. Due to the difficulty in treatment of breast carcinoma and the importance of G2/M checkpoint for cancer cell survival, we speculate that PKMYT1 may be an attractive molecular target for treatment of breast cancer.

More importantly, breast cancer is a heterogeneous disease with subtype‐dependent histopathological features and clinical manifestations. TNBC is a unique subtype of breast cancer with a poor prognosis and patients with TNBC have higher risks of relapse. Due to the lack of therapeutic targets, patients with TNBC are unable to benefit from endocrine therapy or HER2‐targeted therapy, which is the current mainstay of adjuvant therapy. Furthermore, patients with TNBC are more likely to develop chemoresistance. As shown by our study, high expression of PKMYT1 largely predicts the unfavourable prognosis in TBNC with shorter period of RFS. Thus, targeting PKMYT1 may be a promising strategy for therapeutic intervention against TNBC.

Previous study has suggested a potential link between PKMYT1 and β‐catenin/TCF signalling as shown by downregulation of β‐catenin signalling via PKMYT1 depletion in human derived hepatoma HuH‐6 cells.^44^ β‐catenin/TCF signalling is known to be a driving force of EMT in various cancers.^46^ Several major EMT modulators (twist, snail, slug, etc) are target genes for β‐catenin/TCF signalling.^47^, ^48^ Given that EMT is a key limiting step in metastasis,^49^ targeting β‐catenin/TCF signalling via PLMYT1 inhibition may be a promising strategy for cancer therapy.

Polo‐like kinase (PLK1), a key regulatory kinase involved in mitosis and cell cycle progression,^50^, ^51^ plays an important role in tumour cell anabolism by activating the pentose phosphate pathway.^52^ The positive correlation of PLK1 and PKMYT1 in cancer cells may indicate a particular G2 checkpoint mechanism which synchronizes the rapid cell proliferation in accordance with maintenance of genomic stability. Mechanistically, PKMYT1 is highly expressed in cancer cells, and G2/M check is performed to ensure genomic stability. Simultaneously, the duration for G2/M checkpoint should be precisely controlled by PLK1 regulatory pathway for rapid cell proliferation. Co‐targeting these two collaborative kinases might be an efficient way to treat breast carcinoma.

In summary, we have confirmed the up‐regulation of PKMYT1 and its partner, PLK1, in breast cancer and validated their importance as prognostic factors. We propose that PKMYT1 could be a promising molecular target for the diagnosis and treatment of breast cancer.

## CONFLICT OF INTEREST

All authors declare that they have no conflict of interest.

## AUTHOR’S CONTRIBUTIONS

WY conceived and designed the work. WY, YL and JQ developed the experimental methodologies. YL, JQ, ZD, JH, LL and WY performed the analysis and interpreted the data. WY, YL, ZD, LL, HD, HW discussed the results and wrote the paper. And WY supervised the study. All authors approved the submission of this manuscript.

## Data Availability

Source data of this study were derived from the public repositories, as indicated in the section of “Materials and Methods” of the manuscript. And all data that support the findings of this study are available from the corresponding author upon reasonable request.
